# Bootstrap quantification of estimation uncertainties in network degree distributions

**DOI:** 10.1038/s41598-017-05885-x

**Published:** 2017-07-19

**Authors:** Yulia R. Gel, Vyacheslav Lyubchich, L. Leticia Ramirez Ramirez

**Affiliations:** 1 Department of Mathematical Sciences, University of Texas at Dallas, Richardson, Texas 75080 USA; 2 Chesapeake Biological Laboratory, University of Maryland Center for Environmental Science, Solomons, Maryland 20688 USA; 3 Centro de Investigación en Matemáticas, Guanajuato, 36023 Mexico

## Abstract

We propose a new method of nonparametric bootstrap to quantify estimation uncertainties in functions of network degree distribution in large ultra sparse networks. Both network degree distribution and network order are assumed to be unknown. The key idea is based on adaptation of the “blocking” argument, developed for bootstrapping of time series and re-tiling of spatial data, to random networks. We first sample a set of multiple ego networks of varying orders that form a patch, or a network block analogue, and then resample the data within patches. To select an optimal patch size, we develop a new computationally efficient and data-driven cross-validation algorithm. The proposed fast patchwork bootstrap (FPB) methodology further extends the ideas for a case of network mean degree, to inference on a degree distribution. In addition, the FPB is substantially less computationally expensive, requires less information on a graph, and is free from nuisance parameters. In our simulation study, we show that the new bootstrap method outperforms competing approaches by providing sharper and better-calibrated confidence intervals for functions of a network degree distribution than other available approaches, including the cases of networks in an ultra sparse regime. We illustrate the FPB in application to collaboration networks in statistics and computer science and to Wikipedia networks.

## Introduction

Motivated by a plethora of modern large network applications and rapid advances in computing technologies, the area of network modeling is undergoing a vigorous developmental boom, spreading over numerous disciplines, from computer science to engineering to social and health sciences. However, whereas probabilistic models have been dominating the area of network sciences, development of statistical inference, particularly for nonparametric methods for large sparse networks, is noticeably delayed and is still much less investigated^1–5^.

Challenges of parametric model specification and validation for graph-structured data inspire a recent spike of interest in more data-driven and flexible nonparametric (at least, semiparametric) approaches for network inference. As Freno *et al*.^6^ state, “*statistical modeling of networks cries for nonparametric estimation*, *because of the inaccuracy often resulting from fallacious parametric assumptions*”. In spite of that, the scope and availability of nonparametric procedures for random network inference still remains very limited and scarce (for some recent results and overview see refs 3, 7–9 and references therein). In this light, it is appealing and promising to follow a nonparametric bootstrap path for statistical inference on random networks that can potentially allow us to avoid many restrictive conditions on network degree distribution and model specification. To our knowledge, the pioneers in this area are Snijders and Borgatti^10^ who proposed to employ an induced graph sampling for estimation of standard errors in network density estimation and comparison of two networks. The procedure is, however, limited to small networks, assumes availability of the entire network data upfront as well as requires resampling of the entire data set.

Despite all the recent interest in nonparametric network analysis, bootstrap methodology for inference on random networks remains virtually unexplored. And, whereas some recent results target quantification of estimation accuracy for subgraph patterns^8, 11^ and application of bootstrap for community detection^12, 13^, issues with reliable evaluation of estimation errors for a degree distribution are largely unaddressed^14^. Thompson *et al*.^9^ propose a nonparametric resampling-based patchwork bootstrap, with a focus on a network mean degree. In this paper, we further advance the patchwork approach of Thompson *et al*.^9^ and develop a fast and information greedy bootstrap for quantification of estimation uncertainties in functions of degree distribution. To our knowledge, the proposed approach is the first attempt to quantify estimation uncertainty in degree distribution using nonparametric bootstrap. Our framework is different from the most currently available settings in a number of ways. First, we do not hypothesize a particular network model structure. Second, we assume that there exists only a single realization of a network of potentially increasing order. This is in contrast to impractical but conventional assumption that there are multiple available independent network realizations. (In reality, there exists just one Facebook or LinkedIn network). Third, our goal is to utilize only a small portion of observed graph-structured data.

Our idea behind the bootstrap path is intuitive: as the classical bootstrap of Efron^15^ was originally suggested for independent and identically distributed data and then adapted to time series and spatial processes^16–19^, we borrow the “blocking” argument developed for resampling of space and time dependent processes and adjust it to networks. In this sense, a random graph can be viewed as a mathematical object representing a hybrid of time and space dependent processes, with a natural metric induced by a shortest path between two vertices. Similar to the “blocking” argument, we select multiple ego-networks, that is, local vicinities, or patches, around randomly selected vertices (egos), and then resample vertices within each patch. Since patches are allowed to overlap, our procedure can be said to follow the “Künsch rule”^18^. In contrast to the classical “blocking” argument in time series, we do not aim to reconstruct the network data generating process (DGP). Although such DGP reconstruction would certainly be desirable, we believe that this ambitious goal cannot be attained with the patchwork bootstrap or any other bootstrap technique on networks without imposing very restrictive (thus, impractical) conditions on the network structure.

In this paper, we apply the new fast patchwork bootstrap (FPB) to quantify estimation uncertainty in network degree distribution, i.e., develop a confidence interval, under the assumption that both network degree distribution and network order are unknown. Moreover, we explore utility of FPB for ultra sparse networks, that is, the mean degree is constant while the network order increases.

We also found that the new information-greedy bootstrap procedure is sensitive to the size of the patch, similarly to the block bootstrap for space and time dependent data^20^. We address this issue by developing a data-driven and computationally efficient optimal patch selection algorithm based on a cross-validation argument.

The main contributions of our study are as follows:

- To our knowledge, this is the first approach to developing bootstrap inference and bootstrap confidence intervals for network degree distribution. In fact, while there exists a vast literature on graph sampling for estimating network properties (see, e.g., overviews^11, 14, 21^ and references therein), very little is known on how to *reliably evaluate associated errors of estimation* (outside of extensive, information costly, and typically impractical simple random sampling).
- We introduce a novel nonparametric bootstrap method for evaluating uncertainty in functions of a population network degree distribution, under no prior information on network degree distribution and network order. Note that this is very different from developing a point estimator of a quantity of interest, as our new method enables us to assess the error of estimation and construct reliable confidence intervals in a fully data-driven way. Moreover, in contrast with other methods, the network can be ultra sparse and can be only partially observable.
- We develop a new computationally efficient and data-driven cross-validation algorithm for selecting an optimal patch size.
- We validate the new bootstrap procedure by extensive simulations and show that the new method outperforms the competing approaches by providing sharper and better-calibrated confidence intervals for functions of a network degree distribution. We illustrate utility of the FPB in applications to the collaboration and Wikipedia networks.
- Our method allows to draw statistical inference about the “true” (population) unobserved network, using only a small portion of observed graph.
  A short non-archival version of this paper was presented at the 12th SIGKDD Workshop on Mining and Learningwith Graphs^22^.

## Background and Approach

### Assumptions

Consider an undirected random graph *G* = (*V*, *E*) with a set of vertices, *V*(*G*), and a set of edges, *E*(*G*). The order and size of *G* are defined as the number of vertices and edges in *G*, i.e., |*V*(*G*)| and |*E*(*G*)|, respectively (|·| denotes cardinality of a set). We assume that *G* has no self-loops, i.e., *u* ≠ *v* for any edge *e*_*u*v_ ∈ *E*. The degree of a vertex *v* is the number of edges incident to *v*. We denote the probability that a randomly selected node has a degree *k* by *f*(*k*), the degree distribution of *G* by $F=\{f(k),k\ge \mathrm{0\}}$, and the mean degree of *G* by *μ*(*G*). We assume that *G* is involution invariant^23, 24^, that is from the vantage point of any randomly selected vertex, the rest of the connected network is probabilistically the same.

Graph *G* represents some hypothetical “true” random graph of interest that is never fully observed, its order and degree distribution *F* with finite mean are unknown. Instead, we observe a random graph *G*_*n*_ of order *n* with degree distribution ${F}_{n}=\{{f}_{n}(k),k\ge 0\}$. Let ${N}_{k}^{(n)}$ be the number of vertices with a degree *k* in *G*_*n*_. Observed graph *G*_*n*_ is a realization of *G* in a sense that as *n* → ∞, ${N}_{k}^{(n)}/n\to f(k)$ in probability (empirical distribution *F*_*n*_ converges in probability to *F*) and joint degree distribution of *G*_*n*_ approaches that of *G* (see refs 25, 26 and references therein).

### Fast patchwork bootstrap (FPB)

We develop a new nonparametric bootstrap-based inference for an unknown population degree distribution *F* of *G* using the observed realization *G*_*n*_. Let *η*(*G*) be the statistical parameter of interest based on *F* (e.g., *η*(*G*) can be a probability of observing a vertex of degree *k*, network mean degree, variance or tail indexes) and let $\hat{\eta }({G}_{n})$ be an empirical estimator of *η*(*G*) obtained from an observed realization *G*_*n*_. Our goal is to assess estimation uncertainty of the population parameter *η*(*G*) using a bootstrap distribution of the sample statistic $\hat{\eta }({G}_{n})$.

Our patchwork algorithm consists of two main steps: *sampling*, or creation of patches (multiple ego-networks) that aim to “mirror” *G*_*n*_, and *resampling*, or bootstrap, within the patches that aims to quantify estimation uncertainty of the parameter of interest, *η*(*G*). This new method significantly extends and simplifies the approach of Thompson *et al*.^10^, particularly, excludes any nuisance parameters from constructing confidence intervals and does not assume independence of patches.

#### Sampling-resampling procedure is summarized in Algorithm 1

**Figure 1:**
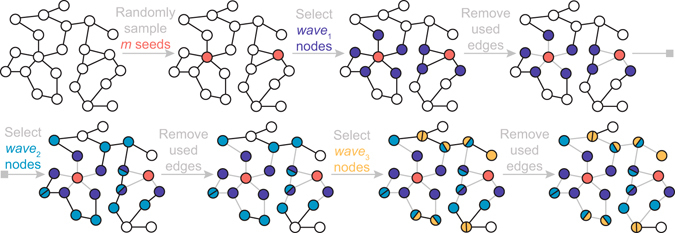
Steps of the LSMI algorithm with *m* = 2 seeds and *d* = 3 waves applied to a network of order *n* = 23.

To generate patches, we employ a modified version of snowball sampling, namely the Labeled Snowball with Multiple Inclusions (LSMI, Fig. 1) of Thompson *et al*.^10^. Algorithm 1 operates with *seeds* (nodes randomly sampled from a network) and *waves* (nodes reached at the *j*th step of growing a snowball around each seed). Unlike snowball sampling, LSMI incorporates new information from the waves conditionally on the links that have been already recorded, thus, does not trace the same edge multiple times and hence minimizes bias in degree estimation. LSMI may be viewed as a fusion of classical snowball sampling, induced subgraph sampling and star sampling^27, 28^.
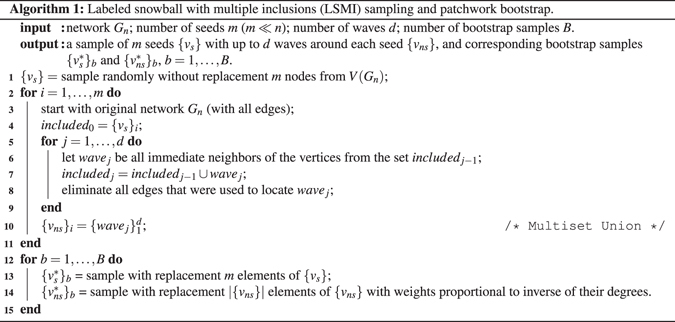

We apply a modified bootstrap-based Horvitz–Thompson method to obtain bootstrap estimates of a degree distribution^10^:

1 $${\hat{f}}^{\ast }(k)=\frac{|\{{v}_{s}^{\ast }(k)\}|+(1-{\hat{p}}_{0}^{\ast })|\{{v}_{ns}^{\ast }(k)\}|}{|\{{v}_{s}^{\ast }\}|+|\{{v}_{ns}^{\ast }\}|},$$

where ${v}_{s}^{\ast }(k)$ and ${v}_{ns}^{\ast }(k)$ are bootstrapped seeds and non-seeds with degree *k*, *k* > 0, ${\hat{p}}_{0}^{\ast }$ is the proportion of zeros in the set of bootstrapped seeds $\{{v}_{s}^{\ast }\}$, and ${\hat{f}}^{\ast }(0)={\hat{p}}_{0}^{\ast }$. The corresponding bootstrap-based mean degree estimator is:

2 $$\hat{\mu }{({G}_{n})}^{\ast }=\sum _{k\ge 0}k{\hat{f}}^{\ast }(k)=\frac{{\hat{E}}^{\ast }(k)|\{{v}_{s}^{\ast }(k)\}|+\mathrm{(1}-{\hat{p}}_{0}^{\ast }){\sum }_{k\ge 1}k|\{{v}_{ns}^{\ast }(k)\}|}{|\{{v}_{s}^{\ast }\}|+|\{{v}_{ns}^{\ast }\}|},$$

where ${\hat{E}}^{\ast }(k)={\sum }_{k\ge 0}k|\{{v}_{s}^{\ast }(k)\}|/|\{{v}_{s}^{\ast }\}|$, i.e., the bootstrap mean degree estimator based solely on seeds. The intuitive idea behind equation (1) is that its numerator represents an estimate of the number of all nodes with a degree *k*, with the first term delivering information from seeds and the second term delivering information from non-seeds. Denominator in equation (1) is an estimator of a network order and, similarly, is based on seeds and non-seeds.

For each seed-wave combination *j* (combination of the number of seeds and number of waves), we construct the Efron 100(1 − *α*)% bootstrap confidence interval

3 $$BC{I}_{j}=({\hat{\eta }}_{[B\alpha /2]}^{j\ast },{\hat{\eta }}_{[B(1-\alpha /2)]}^{j\ast }),$$

where *j* = 1, …, *J*, *J* = *ld*, *d* is the number of waves, *m*_1_, …, *m*_l_ are different sample sizes for the seeds, ${\hat{\eta }}_{[B\alpha /2]}^{j\ast }$ and ${\hat{\eta }}_{[B\mathrm{(1}-\alpha \mathrm{/2)]}}^{j\ast }$ are the empirical quantiles from the bootstrap distribution based on *B* bootstrap replications (see below on a data-driven choice of the optimal seed-wave combination). Throughout the paper, we co﻿nsider a﻿ nominal significance level *α* of 0.05.

#### What do we gain by combining seeds and non-seeds into a joint estimator?

**Figure 2:**
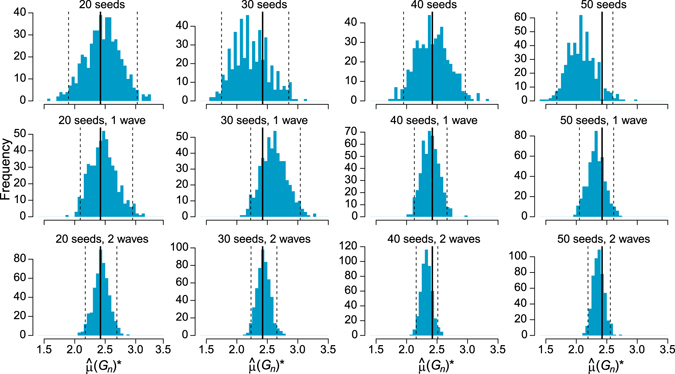
Histograms of bootstrap mean degrees $\hat{\mu }{({G}_{n})}^{\ast }$ for a simulated network of order 10,000 with polylogarithmic(0.1, 2) degree distribution. The 95% confidence intervals (dashed vertical lines) are for *μ*(*G*) = 2.42 (solid vertical lines).

While many estimators of graph totals based solely on seeds are unbiased^29^, variance of such seed-based estimators might be high if the number of seeds is low. At the same time, sampling more seeds might be prohibitively expensive (see overview^30^ and references therein). Adding information from non-seeds into the degree estimator increases bias but reduces variance. Figure 2 demonstrates the effect of adding waves of non-seeds into the mean degree estimator (2). Hence, a choice on number of seeds (egos) and waves of non-seeds in LSMI leads to a classical bias vs. variance trade-off, and we propose to address it using a cross-validation procedure.

#### Asymptotic properties

Let *G*_*n*_ be the observed network and $\hat{f}(k)$ be the estimator of the degree distribution, based on the LSMI sampling of *G*_*n*_. Then, our goal is to show that given *G*_*n*_, the limiting distributions of $\hat{\eta }({G}_{n})$, based on $\hat{f}(k)$, and the bootstrap estimator $\hat{\eta }{({G}_{n})}^{\ast }$, based on (1), coincide. However, a formal theoretical statement on consistency requires derivation of variance of a (non-bootstrap) degree estimator $\hat{f}(k)$, whereas variance of graph totals in snowball sampling is intractable in a closed form beyond first wave^29, 31^. Below we state a conjecture and sketch how the formal consistency proof can be approached.

**Proposition 1**. *Let G be a hypothetical undirected involution invariant network with the degree distribution* $F=\{f(k),k\ge 0\}$ *such that F has finite fourth moment*. *Suppose that our parameter of interest*, *η*(*G*), *is a network mean degree μ*(*G*). *Consider a sequence of observed random networks* $\{{G}_{{n}_{1}},{G}_{{n}_{2}},\ldots ,{G}_{{n}_{j}},\ldots \}$ *and a sequence of numbers of sampled seeds* {*m*_1_, *m*_2_, …, *m*_*j*_, …} *in the patchwork bootstrap algorithm*, *where* *n*_*j*_ *and* *m*_*j*_ *both increase as j* → ∞ *and m*_*j*_/*n*_*j*_ → 0. *(For simplicity*, *we further suppress the index j)*. *Suppose that the highest considered wave in the patchwork bootstrap algorithm is d and upon sampling a seed*, *the network is observable up to* 2*d* − 1 *waves*. *Let* ${F}_{n}=\{{f}_{n}(k\mathrm{),0}\le k\le n\}$ *be a degree distribution of G*_*n*_ *and let G and G*_*n*_ *satisfy the assumptions above*. *Let P*_*n*_ *be the probability function for* $\sqrt{n}(\hat{\mu }({G}_{n})-\mu (G))$, *and* ${P}_{n}^{\ast }$ *be the conditional probability function for* $\sqrt{n}(\hat{\mu }{({G}_{n})}^{\ast }-{E}^{\ast }\hat{\mu }{({G}_{n})}^{\ast })$, *given G*_*n*_. *If*

4 $$n{{\rm{Var}}}^{\ast }(\hat{\mu }{({G}_{n})}^{\ast })-n{\rm{Var}}(\hat{\mu }({G}_{n}))\to 0$$

*in probability*, *then as n* → ∞, *m* → ∞ *and m*/*n* → 0

5 $$\rho ({P}_{n}^{\ast },{P}_{n})\to 0$$

*in probability*, *where ρ is some suitable distance metric between two distributions*.

See Supplementary Information for a justification of Proposition 1.

## Selecting an optimal seed-wave combination

Similar to findings for block bootstrap for space and time dependent processes^17–19, 21^, performance of the new FPB procedure strongly depends on the size of patches defined by the number of seeds (egos) and the number of waves in a patch. We propose to select an optimal combination of these numbers (seed-wave combination) by a data-driven cross-validation procedure (Algorithm 2). Note that in contrast to the earlier method^10^, which requires multiple LSMIs (≈25), the new cross-validation Algorithm 2 requires substantially less data and is based on one LSMI, which makes it particularly attractive for streaming applications.
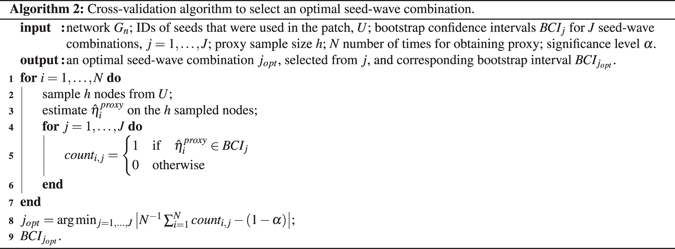


## Simulation study

In this section, we examine finite sample properties of the new fast patchwork bootstrap and cross-validation procedure, by extensive Monte Carlo experiments.

### Validation Metrics

We use two standard statistical metrics to validate the proposed bootstrap method: coverage probability and sharpness. Coverage probability for a 100(1 − *α*)% confidence interval (CI) is defined by a relative proportion of times when the confidence interval contains the estimated parameter. Coverage probability is a measure of *calibration*. Average width of the developed CIs provides assessment of *sharpness*. Calibrated CIs with shorter widths are preferred. Conservative CIs (over-estimating coverage) are preferred over liberal CIs (under-estimating coverage).

**Figure 3:**
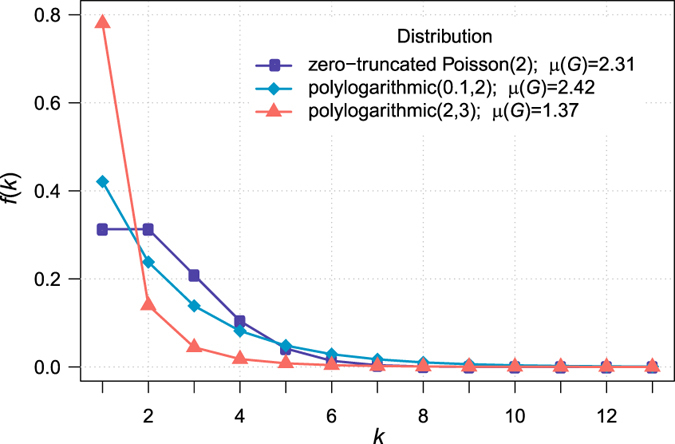
Theoretical degree distributions.

Using the *pairing model* (also known as the *configuration model*) as described by Molloy and Reed^32^ and a rewiring process, that allows to generate a synthetic simple graph with a given degree sequence, including a class of power-law degree distributions^33–35^, we simulate 10,000 networks for three different distributions, namely, zero-truncated Poisson and two different polylogarithmic distributions^10, 36^, and for varying network orders (1,000, 3,000, 5,000, and 10,000 vertices). Among the considered degree distributions, polylogarithmic distribution with parameters (2,3) exhibits the lightest tail, whereas the longest tail belongs to polylogarithmic distribution with parameters (0.1,2) (Fig. 3). We consider patches with 20, 30, 40, and 50 seeds and 1 to 5 waves around each seed (patches of *J* = 20 different seed-wave combinations are sampled from each network realization).

We validate our FPB procedure against two competing procedures. The first competing approach is a 100(1 − *α*)% parametric CI based on normal distribution. That is, using simple random sampling (SRS) without replacement, we select *M* nodes and estimate proportion of nodes with degree *k*, i.e., $\hat{f}(k)$. Then, normality-based confidence interval (NCI) based on the *M* nodes sampled from a graph *G*_*n*_ is $NC{I}^{\{M\}}=\hat{f}(k)\pm z{\hat{\sigma }}_{\hat{f}(k)}$, where *z* is the upper *α*/2 point of the standard normal distribution, and an unbiased estimate of the sample variance of $\hat{f}(k)$ ^37^:

$${\hat{\sigma }}_{\hat{f}(k)}^{2}=(\frac{n-M}{n})\frac{\hat{f}(k)(1-\hat{f}(k))}{M-1}.$$

The second competing approach is a nonparametric quantile-based bootstrapped confidence interval (QCI) based on the *M* nodes from SRS. In particular, we resample with replacement the degrees of *M* previously selected nodes, calculate the respective proportions of nodes with degree *k* and repeat the resampling procedure *B* times. The respective Efron bootstrap confidence interval is given by

6 $$QC{I}^{\{M\ast \}}=({\hat{f}}_{[B\alpha /2]}^{\{M\ast \}}(k),\,{\hat{f}}_{[B(1-\alpha /2)]}^{\{M\ast \}}(k)),$$

where ${\hat{f}}_{[B\alpha /2]}^{\{M\ast \}}(k)$ and ${\hat{f}}_{[B(1-\alpha /2)]}^{\{M\ast \}}(k)$ are the empirical quantiles estimated solely from the *M* nodes from SRS.

### Quantifying estimation uncertainty for probabilities *f*(*k*) of observing a node of degree *k*

We now apply the FPB to quantify uncertainty in estimating theoretical probabilities *f*(*k*), *k* ∈ *Z* ^+^, in a ultra sparse regime. That is, network mean degree is fixed and number of vertices *n* increases.

Table 1 presents the results of the new FPB procedure along with the competing NCI and QCI. The FPB provides the most calibrated and sharp confidence intervals for all considered degree distributions and network orders. In particular, for the zero-truncated Poisson distribution and polylogarithmic distribution with parameters (0.1,2), coverage of the FPB fluctuates around the declared 95% confidence level (coverage is between 92% and 98%), while both NCI and QCI, despite consistently yielding around 40% wider intervals than FPB, noticeably underestimate the nominal coverage probability, especially for *f*(4) and *f*(5).

**Table 1:** Coverage of theoretical probabilities *f*(*k*) of observing a node of degree *k*, *k* = 2, …, 5, by 95% confidence intervals for varying network orders.

Degree distribution	*k*	Method	Network order *n*			
			2,000	3,000	5,000	10,000
Zero-truncated Poisson(2)	2	FPB	92.4 (0.15)	93.3 (0.15)	93.7 (0.16)	94.7 (0.15)
		NCI^{50}^	93.0 (0.25)	92.6 (0.25)	92.0 (0.26)	93.2 (0.26)
		QCI^{50*}^	93.5 (0.25)	94.5 (0.25)	92.9 (0.25)	94.4 (0.25)
	3	FPB	94.9 (0.13)	96.2 (0.13)	92.9 (0.13)	95.8 (0.13)
		NCI^{50}^	93.4 (0.22)	95.3 (0.22)	95.1 (0.22)	94.6 (0.22)
		QCI^{50*}^	92.2 (0.22)	93.2 (0.22)	93.5 (0.22)	93.0 (0.22)
	4	FPB	96.4 (0.10)	97.3 (0.10)	97.9 (0.10)	97.7 (0.10)
		NCI^{50}^	89.5 (0.17)	88.7 (0.17)	89.8 (0.17)	89.8 (0.17)
		QCI^{50*}^	90.0 (0.16)	89.6 (0.16)	90.0 (0.16)	89.1 (0.16)
	5	FPB	98.0 (0.06)	97.8 (0.06)	98.9 (0.06)	98.4 (0.06)
		NCI^{50}^	87.9 (0.10)	86.7 (0.10)	86.1 (0.10)	85.9 (0.10)
		QCI^{50*}^	88.0 (0.09)	86.7 (0.09)	86.2 (0.09)	85.9 (0.09)
polylogarithmic(0.1,2)	2	FPB	92.2 (0.13)	92.5 (0.13)	92.3 (0.14)	94.0 (0.13)
		NCI^{50}^	91.5 (0.23)	90.8 (0.23)	91.6 (0.23)	91.8 (0.24)
		QCI^{50*}^	93.8 (0.23)	93.6 (0.23)	94.5 (0.23)	93.5 (0.23)
	3	FPB	92.5 (0.11)	95.3 (0.11)	96.6 (0.11)	96.0 (0.11)
		NCI^{50}^	93.6 (0.19)	91.7 (0.19)	91.5 (0.19)	91.7 (0.19)
		QCI^{50*}^	93.6 (0.19)	91.7 (0.18)	91.7 (0.18)	92.9 (0.18)
	4	FPB	93.9 (0.08)	96.5 (0.08)	96.7 (0.09)	98.2 (0.09)
		NCI^{50}^	90.0 (0.14)	91.4 (0.15)	90.9 (0.15)	93.4 (0.15)
		QCI^{50*}^	89.9 (0.14)	91.4 (0.14)	90.9 (0.14)	93.3 (0.14)
	5	FPB	97.3 (0.07)	96.8 (0.06)	98.0 (0.06)	98.7 (0.07)
		NCI^{50}^	93.2 (0.11)	92.1 (0.11)	92.1 (0.11)	91.8 (0.11)
		QCI^{50*}^	92.5 (0.10)	91.6 (0.10)	91.9 (0.10)	91.5 (0.10)
polylogarithmic(2,3)	2	FPB	96.0 (0.13)	95.1 (0.13)	95.8 (0.13)	96.7 (0.14)
		NCI^{50}^	89.9 (0.19)	92.7 (0.19)	92.0 (0.19)	90.7 (0.19)
		QCI^{50*}^	90.6 (0.18)	93.3 (0.19)	93.0 (0.18)	92.7 (0.18)
	3	FPB	96.0 (0.08)	96.0 (0.08)	98.6 (0.08)	97.3 (0.08)
		NCI^{50}^	90.1 (0.10)	89.2 (0.10)	88.8 (0.10)	90.5 (0.11)
		QCI^{50*}^	89.7 (0.10)	88.7 (0.10)	88.7 (0.10)	89.7 (0.10)
	4	FPB	96.8 (0.05)	95.8 (0.05)	95.6 (0.05)	96.1 (0.05)
		NCI^{50}^	59.3 (0.06)	58.7 (0.06)	59.4 (0.06)	60.6 (0.06)
		QCI^{50*}^	58.2 (0.05)	57.1 (0.05)	58.2 (0.05)	59.5 (0.05)
	5	FPB	86.7 (0.03)	87.0 (0.03)	86.8 (0.03)	86.2 (0.03)
		NCI^{50}^	33.4 (0.03)	34.8 (0.03)	32.5 (0.03)	34.2 (0.03)
		QCI^{50*}^	33.2 (0.02)	34.8 (0.02)	32.2 (0.02)	34.0 (0.02)

Average interval width is given in parentheses. Methods of obtaining confidence intervals are fast patchwork bootstrap (FPB), normal interval based on estimated proportions and their variance using 50 random nodes (NCI^{50}^), and bootstrap of 50 random nodes (QCI^{50*}^). Number of bootstrap resamples is 500. Number of Monte Carlo simulations is 1,000.

Moreover, difference in performance among the FPB, NCI, and QCI is particularly striking for the sparsest network (polylogarithmic degree distribution with parameters (2,3)). Here, the FPB delivers well calibrated intervals for *f*(2) to *f*(4), closely resembling the declared 95% confidence level; however, despite producing noticeably wider intervals, NCI and QCI cover the true *f*(4) only in 60% of the times under the declared 95% level. While all methods deliver liberal confidence intervals for *f*(5), performance of the FPB is still strikingly better. That is, the FPB-based CIs contain the true *f*(5) value in 86–87% of the cases under the declared 95% confidence, while NCI and QCI contain the true *f*(5) value at most in 35% of the cases.

Thus, the FPB can be viewed as a preferred procedure for fast and reliable inference in even ultra sparse networks, under limited prior information. Moreover, the FPB method is both computationally efficient and information-greedy (i.e., it minimizes information that is collected from the network). Hence, the FPB approach can be of particular importance in analysis of complex social networks, for example, for quantifying estimation uncertainty and hypothesis testing for number of friends, collaborators, and sexual partners, including hard-to-reach populations.

## Case Studies

### Collaboration Networks in Statistics and Computer Science

We illustrate the FPB algorithm in application to analysis of collaboration networks in statistical and computer sciences. Differences in collaboration patterns for various scientific disciplines have been analyzed in numerous studies^36, 38–43^. Analysis of collaboration structure of statisticians is still, however, underexplored^44^. Recently, Coccia and Wang^41^ showed in a non-network setting that average intensity of international collaborations in mathematics (including statistics) and computer science increased at a similar rate, as well as both of these fields exhibit a similar average intensity of collaborations. In view of the recent data science boom and a vanishing borderline between statistics and machine learning disciplines, an interesting question arises whether statisticians and computer scientists exhibit similar or different collaboration patterns.

In our analysis we focus on how many collaborators statisticians and computer scientists are likely to have, that is, we perform inference on the probabilities of having a certain number of co-authors in each discipline. For computer science, we use a network of authors listed in the DBLP computer science bibliography, particularly, the largest connected component^45^. The network of statisticians consists of authors from four journals that are among the top in the field^44^. In both networks, vertices represent authors; edges indicate presence of at least one co-authored paper. To make the networks more comparable, we remove isolated vertices from the network of statisticians, so *f*(0) = 0 in both networks.

Table 2 shows summary networks statistics (i.e., network order *n* and observed mean degree $\hat{\mu }$ calculated over the entire co-authorship networks along with observed probabilities of having 1–5 co-authors) and the 95% confidence intervals delivered by FPB and its competitors NCI and QCI. First, notice that NCI and QCI are substantially wider than the FPB confidence intervals. All NCI and QCI overlap for statistics and computer science. Second, NCI and QCI do not always contain the true value (e.g., *f*(4)). In contrast, the FPB yields up to 40% sharper confidence intervals that in all cases contain the true values *f*(1), …, *f*(5). Moreover, the FPB confidence intervals are better centered in terms of containing the true values *f*(1), …, *f*(5).

**Table 2:** The 95% confidence intervals for the population probabilities *f*(*k*) of two collaboration networks.

*k*	$\hat{{\boldsymbol{f}}}{\boldsymbol{(}}{\boldsymbol{k}}{\boldsymbol{)}}$	FPB		NCI^{50}^		QCI^{50*}^	
		Lower	Upper	Lower	Upper	Lower	Upper
Network of co-authors in computer science: *n* = 317,080; $\hat{\mu }\,=\,6.6$							
1	0.136	0.093	0.197	0.104	0.336	0.100	0.340
2	0.186	0.140	0.280	0.029	0.211	0.040	0.220
3	0.157	0.087	0.187	0.057	0.263	0.080	0.271
4	0.111	0.066	0.142	0.000	0.095	0.000	0.100
5	0.081	0.044	0.115	0.004	0.156	0.020	0.160
Network of co-authors in statistics: *n* = 3,453; $\hat{\mu }=3.3$							
1	0.264	0.218	0.354	0.089	0.311	0.100	0.300
2	0.292	0.135	0.311	0.227	0.493	0.240	0.520
3	0.162	0.081	0.243	0.030	0.210	0.040	0.200
4	0.088	0.014	0.122	0.000	0.059	0.000	0.060
5	0.055	0.037	0.148	0.030	0.210	0.040	0.220

Methods of obtaining confidence intervals are fast patchwork bootstrap (FPB), normal interval based on estimated proportions and their variance using 50 random nodes (NCI^{50}^), and bootstrap of 50 random nodes (QCI^{50*}^). In FPB, 12 seed-wave combinations were considered: waves from 1 to 3, seeds 20, 30, 40, and 50. Cross-validation is based on a random selection of 100 seeds 13 times. Number of bootstrap resamples is 500.

**Figure 4:**
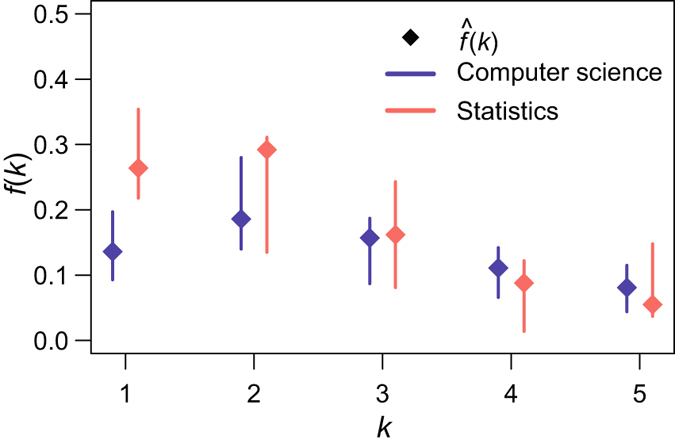
Observed frequencies $\hat{f}(k)$ (points) and FPB 95% intervals (lines) for *f*(*k*), for the two networks of researchers.

Remarkably, while FPB also indicates that the degree distributions of co-authorship in computer science and statistics are overall similar (see Table 2 and Fig. 4), probability of having just a single co-author is different in the two disciplines. Statisticians are twice likelier than computer scientists to collaborate with just one person (i.e., 0.26 vs. 0.13, respectively), and this difference is indeed statistically significant (i.e., the FPB 95%-confidence intervals for *f*(1) in statistics and computer science networks do not overlap). In view of higher reliability of FPB for inference on synthetic networks, we tend to conclude that indeed many more statisticians than computer scientists still work in pairs rather than in larger groups. This phenomenon indicates a still substantial intrinsic influence of mathematical sciences and, particularly, pure mathematics on statistics. For instance, the Oberwolfach Research Institute for Mathematics in Germany and Centre International de Rencontres Mathématiques (CIRM) in France offer a program “Research in Pairs”. Nowadays, both programs are extended to include 2–4 collaborators. However, the program name still inherits “pairs”. A similar but younger institution in Canada, Banff International Research Station for Mathematical Innovation and Discovery, that has arguably a broader focus on both theoretical and applied mathematics, already calls such a program “Research in Teams”.

### Wikipedia Networks

We now show utility of FPB for quantifying estimation uncertainty of mean degree, in application to analysis of Wikipedia networks. Wikipedia is one of the top websites by the size of its multi-platform audience (i.e., desktop, smartphone, and tablet users) in the United States. In October 2016, Wikipedia attracted almost 119 million visitors that corresponded to 46.1% of the total digital population. Moreover, Wikipedia community unites more than 45 million registered users who contribute to more than 31 million articles in 296 languages. Historically, English Wikipedia is substantially bigger than Wikipedias in other languages but in the past few years situation changed dramatically: in November 2005 English Wikipedia accounted for 29.2% of all articles^46^, and in May 2017 its share plummeted to 12.1%.

We are interested in investigating the emerging communities of non-English Wikipedias from a network perspective. As the structure of Wikipedia is complex (it contains articles, categories, images and multimedia, templates, etc.), various networks may arise from the same data^47^. For instance, driven by particular objectives, researchers consider edit networks where nodes represent authors and edges show who delete, undelete, or restore the edits of which authors^48^; analyze categories (nodes) as a semantic space of topics and their similarity values (edges)^46^; focus on articles (nodes) and cross-references (edges) between them^49, 50^; study co-authorship network where Wikipedia users (nodes) are linked together if they are major authors of the same article^51^. In this paper, we aim to compare online activity of Wikipedia users in different languages. Similarly to^52, 53^, we construct four separate social networks (in Hebrew, Italian, Norwegian, and Russian) where nodes stand for talk and user pages while undirected edges represent existence of links between them. The online activity in these networks can be assessed with the network mean degree: the more active the users, the more posts they create on each other’s pages, the more interactions (links) occur. The data on the non-English Wikipedia are collected in the same manner as in earlier studies^49, 53^, including, for example, omitting cross-references among different languages and self-citations, and thus the considered non-English Wikipedia networks are to be viewed as subsets rather than as populations. To account for the uncertainty associated with a sample mean degree, we construct patchwork bootstrap confidence intervals.

Table 3 presents the results of the patchwork bootstrap along with the observed network orders and mean degrees for the four Wikipedia networks. Optimal seed-wave combinations are selected using the cross-validation procedure and for all four networks the optimal patch has one wave around 20, 40, or 50 seeds. The observed mean degree 4.16 of Norwegian Wikipedia turns out to be the lowest among the considered networks followed by the observed mean degree 5.90 of Hebrew Wikipedia. Russian and Italian Wikipedias have the highest observed mean degrees of 8.47 and 9.59, respectively. While the observed mean degree for Hebrew Wikipedia is almost twice smaller than the mean degree of Italian Wikipedia, the obtained 95%-bootstrap confidence intervals for Hebrew, Russian, and Italian Wikipedias overlap, which implies that we fail to reject the null hypothesis of different mean degrees of these networks. In contrast, the bootstrap 95%-confidence interval for Norwegian Wikipedia does not overlap with the respective 95%-bootstrap confidence intervals for Russian and Italian Wikipedias, and hence we are likely to conclude that activity in Norwegian Wikipedia is indeed lower than activity in Russian and Italian Wikipedias. To check the consistency of results, we ran the bootstrap with larger patches of 25, 50, 75, and 100 seeds: even the confidence bounds are volatile, the intervals consistently include the estimated mean degree $\hat{\mu }({G}_{n})$, and the interval for Norwegian Wikipedia does not overlap with intervals for Italian and Russian Wikipedia networks.

**Table 3:** The 95% FPB confidence intervals for the mean degrees of Wikipedia networks, constructed based on the links (edges) between talk and user pages (nodes) in different languages.

Network	*n*	$\hat{{\boldsymbol{\mu }}}({{\boldsymbol{G}}}_{{\boldsymbol{n}}})$	Optimal combination		95% confidence bounds for the mean degree *μ*(*G*)	
			Seeds	Waves	Lower	Upper
Hebrew	7,856,666	5.90	40	1	2.70	9.82
Italian	25,951,119	9.59	20	1	7.97	17.56
Norwegian	3,824,079	4.16	50	1	1.33	6.11
Russian	19,415,432	8.47	40	1	6.26	15.33

The analysis considered 12 seed-wave combinations: seeds 20, 30, 40, and 50; waves from 1 to 3, ﻿and 500 bootstrap resamples per each combination. Cross-validation is based on a random selection of 10 seeds 10 times.

While as might be expected, mean degree in Wikipedias is related to a network order (i.e., larger Wikipedia networks tend to have a higher mean degree), it remains unclear why we observe such differences among non-English Wikipedias. The first possible route is to compare the number of native speakers of each language, which results in about 5 million native Norwegian and Hebrew speakers and about 60 and 160 million of native Italian and Russian speakers, respectively. While the number of native Italian speakers is almost three times lower than the number of native Russian speakers, the network order of Italian Wikipedia is larger than the Russian one, mean degrees for Russian and Italian Wikipedias are almost the same and their respective bootstrap 95%-confidence intervals largely overlap. Hence, the difference in activity among the four non-English Wikipedias cannot be explained by the number of native speakers.

**Figure 5:**
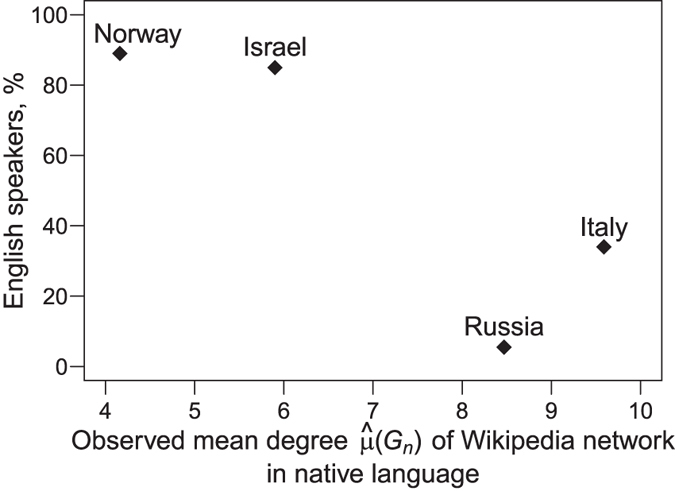
Estimated mean degrees of the Wikipedia networks in Hebrew, Italian, Norwegian, and Russian vs. percent of people in corresponding countries who can speak English.

Remarkably, these findings lead us to the concept of bilinguality^54, 55^. First, notice that proportion of people in Norway, Israel, Russia, and Italy who are proficient in English differ substantially. For instance, Norway has the second highest English Proficiency Index (EPI) of 66.60 in the world and it is closely followed by Israel^56, 57^. In contrast, Russia and Italy have low proficiency in English with EPI of 51.08 and 50.97, respectively. Wikipedia users who are not native English speakers pay the cost of having to learn English as a second language to get additional benefits from the information resources of English Wikipedia and communications with other English speakers. According to the bilinguality hypothesis^54, 55^, the substantial benefits allow the foreign language (in this case, English) to persist in the network and to keep the native language from taking over the rest of the network. Hence, such bilinguality leads to a less developed network in a native language. Figure 5 confirms this conclusion as it shows strong negative correlation between mean degree of Wikipedia networks and the percentage of people with high proficiency in English.

Other possible explanations of heterogeneous mean degrees in Wikipedia’s communities relate to different popularity of this website across the countries and willingness of people to volunteer and contribute to the development of Wikipedia pages.

Overall, the Wikipedia users posting in Norwegian are less active than the users posting in Italian and Russian. However, it does not imply that people from Norway are less active Wikipedia users in general: unlike Russians or Italians, they can easily use Wikipedia in English language, which is prevailing in this online community. In the future, we expect to see further rapid growth of non-English Wikipedia contributed by people from the countries with a relatively low English Proficiency Index (e.g., Mexico, Turkey, and African countries).

## Conclusions

In this paper, we propose a novel data-driven and computationally efficient method for quantifying uncertainty in network degree distribution using nonparametric bootstrap. We primarily focus on developing confidence intervals for functions of a network degree distribution of some “true” underlying network and perceive the collected network data as a single realization of this “true” unobserved network. The proposed patchwork idea is intrinsically linked to block bootstrap and re-tiling in space-time processes where patches, or analogues of blocks and tiles, are grown around randomly selected seeds, and then both seeds and their neighbors are resampled. Similarly to resampling procedures for weakly dependent space-time processes, finite sample performance of the new FPB depends on number of seeds and waves around them, and we address this challenge by developing a new data-driven cross-validation procedure. We show that the FPB provides well-calibrated and sharp confidence intervals for network mean degree and probabilities of observing a node of a prespecified degree and outperforms its parametric and nonparametric competitors in terms of accuracy, computational costs, and required network information. The current version of the FPB code is available from R package *snowboot* ^58^.

The new bootstrap method can be further extended to quantification of estimation uncertainty in point centrality and centralization measures, network heterogeneity and similarity measures for multiple network comparisons based on a degree distribution. Note that performance of any sampling estimator for a degree distribution (and in fact, of other network statistics as well) depends on a fraction of utilized graph data^22^. Hence, there always exists a limitation on how well we can estimate a tail of the degree distribution, given a fixed proportion of observed network information. Hence, we believe that combining our bootstrap approach for quantification of estimation uncertainties with an inverse method^22^ for estimating degree distribution, might reap the benefits of the two worlds. That is, we can first study the linkage between the inverse point estimator^22^ and bootstrap distributions for lower degrees for which we can have both the point estimators^22^ and the respective bootstrap distributions. Then we propagate uncertainty quantification by extrapolating bootstrap estimates across higher degrees, for which we have only the inverse point estimator^22^. The proposed bootstrap methodology can be also extended to a case of directed networks, that is, for nonparametric inference on functions of in-degree and out-degree distributions. This extension will primarily affect the choice of a sampling design that is more suitable for directed networks. For instance, instead of a snowball sampling we can use random walks on directed networks and then resample non-seed nodes with weights, proportional to inverse of their in- and out-degrees. Furthermore, instead of the modified Horvitz–Thompson estimator (1), we can employ estimators adapted to walk sampling and respondent driven sampling (RDS) on directed graphs^28, 59, 60^. Furthermore, the proposed bootstrap approach can be employed to test for a modular structure of a network and for network anomalies under the null hypothesis of no structure, which can be approached, for instance, by comparing bootstrap degree distributions and associated statistics for multiple subnetworks. Another interesting direction is application of bootstrap for goodness-of-fit testing on networks and optimal parameter selection, for instance, in conjunction with parameterization of the shortest-path distance distribution of networks using the generalized Gamma distribution^61^.

Finally, we would like to conclude our paper with the following quote of Snijders and Borgatti from their pioneering paper^11^ on using nonparametric bootstrap for random networks: “the basis for [their] non-parametric standard errors and probabilities is mainly intuitive” but “(a) there seem to be no alternatives in the general case, and (b) it is better to have a rough impression of the uncertainty or variability associated with observed network statistics than none at all. Therefore we hope that especially the bootstrap standard error will be applied widely by network analysts”. Remarkably, not much has been achieved and published on the nonparametric bootstrap direction for random networks since^11^. Hence, in no way our paper can be viewed as a universal and closed-form solution for nonparametric bootstrap inference on random networks but rather as another step on this challenging path to more data-dependent methods for assessing and quantifying network estimation uncertainties.

## Electronic supplementary material

Supplementary Material
