# Supplementary material for: Bootstrap quantification of estimation uncertainties in network degree distributions
Source: Sci Rep. 2017 Jul 19;7:5807. doi: 10.1038/s41598-017-05885-x (PMC5517433; doi:10.1038/s41598-017-05885-x)
Supplement: Supplementary file 1 — Supplementary Material [file 41598_2017_5885_MOESM1_ESM.pdf]

Supplementary material for: Bootstrap quantification of estimation uncertainties in network degree distributions

Yulia R. Gel, Vyacheslav Lyubchich, and L. Leticia Ramirez Ramirez

Proof sketch for Proposition 1

The rationale behind equation (5) is to show that given some optimal selection of patch size, distribution of $\hat{\mu}(G_n) = \sum_{k\geqslant 0} k\hat{f}(k)$ and conditional distribution of $\hat{\mu}(G_n)^* = \sum_{k\geqslant 0} k\hat{f}^*(k)$, given G_n, asymptotically follow a normal distribution with the same mean and standard deviation, which in turn implies equation (5).

Consider first a bootstrap estimator $\hat{\mu}(G_n)^*$. Sampling of seeds in the fast patchwork bootstrap (FPB) algorithm is performed without replacement, but if the number of seeds $m \ll n$, this should be almost equivalent to sampling with replacement. To derive the expected mean and variance of numerator and denominator on the right-hand side of (2), we need to derive expressions for inclusion of a randomly selected vertex as a seed and non-seed. For seeds, the inclusion probability is approximately m/n if $m \ll n$. For non-seeds, the probability of selecting a vertex of degree k as a non-seed in the first wave is $\simeq km/n$. To get approximate expressions for inclusion probabilities for non-seeds in higher waves (up to the d-th wave), we use the condition on partial observability of the network up to $2d-1$ waves and then employ the combinatorial argument of Snijders[1]. Armed with the resulting inclusion probabilities, we then use Theorem 4 of Berger[2], which implies that conditionally on G_n, both numerator and denominator of the term on the right-hand side of equation (2) are asymptotically normally distributed under unequal probability sampling. Finally, we apply the delta method to show that the conditional distribution of the bootstrap estimator of mean degree (2), given G_n, is asymptotically normal[3,4]. Asymptotic normality of the non-bootstrap mean degree estimator $\hat{\mu}(G_n)$ can be approached in a similar manner.

However, bootstrap consistency in equation (5) requires that the bootstrap variance is asymptotically correct, i.e., $n\mathrm{Var}^*(\hat{\mu}(G_n)^*) - n\mathrm{Var}(\hat{\mu}(G_n)) \to 0$ in probability. Echoing the earlier discussion[1,5], it is not possible to derive variances of non-bootstrap ($\hat{\mu}(G_n)$) and bootstrap ($\hat{\mu}(G_n)^*$) estimators in a closed form. One possible theoretical approach in this direction is to employ a branching process approximation up to the d-th wave, which leads, however, to another open question on quantification of the related approximation error.

An alternative heuristic approach is to achieve the desired relationship between variances of $\hat{\mu}(G_n)$ and $\hat{\mu}(G_n)^*$ using optimal patch size selection and cross-validation. That is, using a cross-validation argument we find such optimal number of seeds (egos) m_{opt} and number of waves d_{opt} that

$$\mathrm{Var}^*(\hat{\mu}_{m_{opt},d_{opt}}(G_n)^*)/\mathrm{Var}(\hat{\mu}(G_n)) \approx 1,$$

and this is the method undertaken in this paper.

References

1. Snijders, T. A. B. Estimation on the basis of snowball samples: How to weight? *Bulletin of Sociological Methodology* **36**, 59–70 (1992). DOI 10.1177/075910639203600104.
2. Berger, Y. G. Rate of convergence for asymptotic variance of the Horvitz–Thompson estimator. *Journal of Statistical Planning and Inference* **74**, 149–168 (1998). DOI 10.1016/S0378-3758(98)00107-4.
3. van der Vaart, A. W. *Asymptotic Statistics* (Cambridge University Press, Cambridge, 1998).
4. Prášková, Z. & Sen, P. K. Asymptotics in finite population sampling. In Pfeffermann, D. & Rao, C. R. (eds.) *Sample Surveys: Inference and Analysis*, 489–523 (Elsevier, Amsterdam, 2009).
5. Frank, O. Estimation of graph totals. *Scandinavian Journal of Statistics* **4**, 81–89 (1977).
